# A protocol to investigate the effects of lncRNAs on *in vivo* protein-protein interactions using proximity ligation assay

**DOI:** 10.1016/j.xpro.2023.102757

**Published:** 2023-12-02

**Authors:** Ling Zhang, Mengfan He, Peizhen Wang, Jianfeng Yu, Dawei Li

**Affiliations:** 1 Center for Translational Medicine, The Affiliated Zhangjiagang Hospital of Soochow University, Suzhou Medical College of Soochow University, 68 Jiyang West Road, Suzhou 215600, China; 2 Department of Life Science and Technology, Changshu Institute of Technology, 99 South Third Ring Road, Suzhou 215500, China

**Keywords:** Cell Biology, Cancer, Microscopy, Molecular/Chemical Probes, Protein Biochemistry

## Abstract

A large variety of cellular signals are triggered and transmitted by protein-protein interactions (PPIs). Long noncoding RNAs regulate PPIs by enhancing or destabilizing these interactions. Here, we use the proximity ligation assay technique to determine PPIs between p53 and SET regulated by long intergenic noncoding RNA 324 (LINC00324). We detail procedures for establishing LINC00324 knockdown and overexpression U2OS and HepG2 cells followed by *in situ* PLA protocol. This approach has many potential applications for the study of cellular factors that regulate PPIs.

For complete details on the use and execution of this protocol, please refer to Zhang et al. (2023).^1^

## Highlights

• Preparation of LINC00324 knockdown and overexpression stable cell lines
• Choice of primary antibodies is critical for an efficient PLA
• Assay for detecting the p53-SET interaction *in vivo* using PLA
• LINC00324 destabilizes the p53-SET interaction *in vivo*

## Abstract (editor highlights)

Publisher’s note: Undertaking any experimental protocol requires adherence to local institutional guidelines for laboratory safety and ethics.

## Graphical abstract

**Figure:**
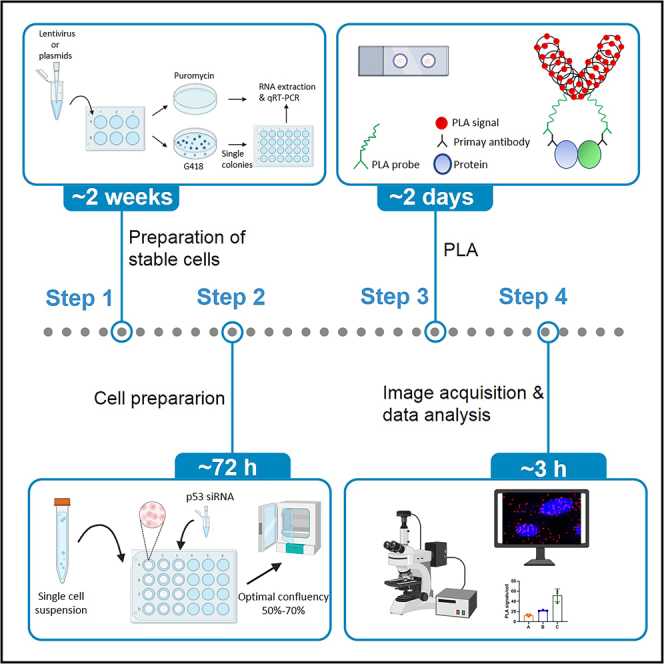

## Before you begin

**Figure 1:**
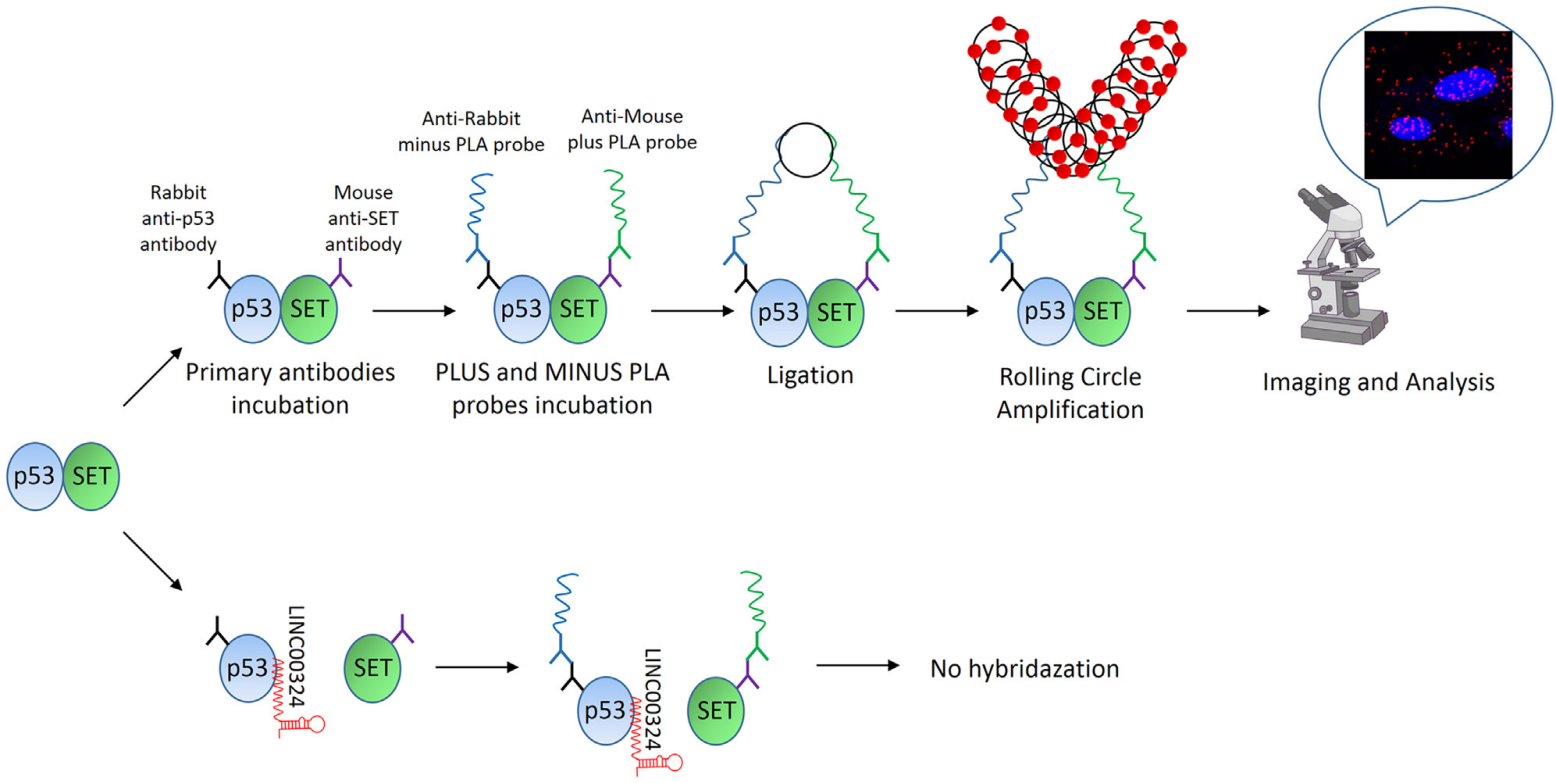
Workflow overview of the Duolink proximity ligation assay (PLA) Step by step representation of PLA detection of p53-SET interaction. LINC00324 disrupts the p53-SET interaction.

There are a variety of methods one can use to investigate PPIs in cells, including immunofluorescence staining, co-immunoprecipitation, fluorescence resonance energy transfer (FRET), and proximity ligation assay (PLA).^2^^,^^3^^,^^4^^,^^5^ Duolink PLA technology allows one to visually recognize PPIs at single cell resolution with high sensitivity and specificity.^6^ Here, we describe a protocol for visualization of p53-SET interaction regulated by LINC00324 (Figure 1).

### Preparation of LINC00324 knockdown and overexpression stable cell lines

**Timing: ∼2 weeks**

The following steps describe the procedure to establish LINC00324 knockdown and overexpression stable cell lines. The knockdown cells were generated using shRNA expression lentivirus with puromycin resistance. The control and LINC00324 shRNA sequences (Table 1) were cloned into lentivirus vector LV2 (U6/Puro). The lentivirus constructs were co-transfected with helper vectors, pGag/Pol, pRev and pVSV-G into 293T packaging cells (GenePharma, Suzhou, China). The cell supernatants were collected into 50 mL centrifuge tubes and centrifuged at a low speed of 400 g for 4 min at 4°C. Then the supernatants were filtered through a 0.45 μm filter. The filtrates were collected by ultra-centrifuging for 2 h at 3000 g at 4°C. For stable ectopic LINC00324 expression, LINC00324 complementary DNA (cDNA) was inserted into the pcDNA3.1/myc-His-A (+) vector to generate LINC00324 overexpression constructs. The empty vector was used as a control (CON). These constructs were transfected into cells using Lipofectamine 2000 transfection reagent (Invitrogen). The stable cells overexpressing LINC00324 were screened by G418 resistance selection.

1. Preparation of LINC00324 knockdown stable cell lines.
  a. The day before infection, seed U2OS and HepG2 cells in a 6-well plate at a density sufficient to reach 50%–60% confluence on the day of infection.
  b. Incubate the cells in incubator at 37°C, 5% CO_2_ for 16–24 h.
  c. Mix the lentivirus expressing control and LINC00324 shRNAs with culture medium in total volume of 2 mL to achieve a multiplicity of infection (MOI) of 50–100 for both cells, add polybrene to a final concentration of 5 μg/mL.
  d. Move out the medium from the 6-well plate, replace with the virus diluent and incubate at 37°C in the CO_2_ incubator for 24 h.
  e. Replace with 2 mL fresh medium and incubate at 37°C in the CO2 incubator for 24–48 h.
  f. Select cells with 2 μg/mL puromycin for 2 weeks. During this process, replace with fresh medium containing 2 μg/mL puromycin every 2–3 days.
  g. Collect (0.5–1)×10^6^ cells from control and knockdown groups, extract cellular total RNAs using TRIzol reagent and then reverse-transcribed into cDNAs.
  h. Verify interference efficiency using quantitative real-time PCR (qRT–PCR) (Figures 2A and 2B).

**Figure 2:**
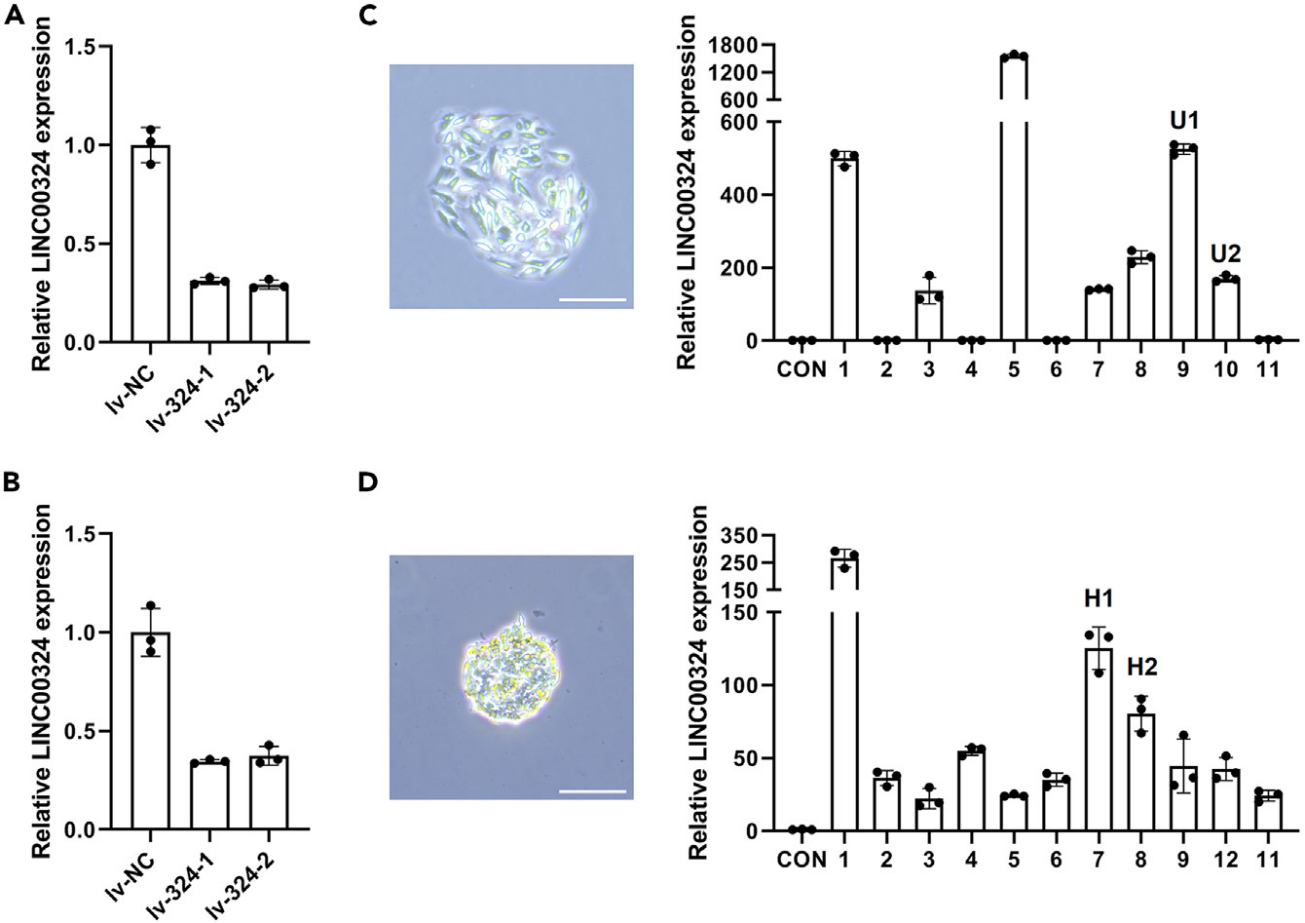
Preparation of LINC00324 knockdown and overexpression U2OS and HepG2 cells (A and B) The expression of LINC00324 was detected by qRT–PCR in LINC00324 knockdown U2OS (A) and HepG2 cells (B). (C and D) Colonies were selected after G418 selection for 2 weeks. qRT–PCR was used to determine the expression of LINC00324 to select positive over-expression cells in U2OS (C) and HepG2 cells (D). Two positive stable cell lines for each cell type were selected for subsequent experiments. Scare bar, 100 μm.
2. Preparation of LINC00324 overexpression stable cell lines.
  a. One day before transfection, plate U2OS and HepG2 cells in a 6-well plate at a density sufficient to reach 70%–90% confluence at the time of transfection.
  b. For each well to be transfected, prepare mixture as follows.
    i. Dilute 5 μg plasmids in 250 μL of Opti-MEM I Reduced Serum Medium.
    ii. Dilute 10 μL Lipofectamine 2000 in 250 μL of Opti-MEM medium. Incubate for 5 min at room temperature (22°C–26°C).
    iii. Gently mix the diluted plasmids with diluted Lipofectamine 2000. Incubate for 20 min at room temperature (22°C–26°C).
  c. Replace the medium in the 6-well plate with 1.5 mL Opti-MEM medium. Add the 500 μL of transfection complexes into each well. Mix gently by rocking the plate back and forth.
  d. Incubate cells at 37°C in the CO_2_ incubator for 4–6 h. Replace the medium containing the transfection reagent with a regular culture medium.
  e. Incubate cells at 37°C in the CO_2_ incubator for 24 h.
  f. After digestion, seed cells at 1:50, 1:100, 1:200, and 1:500 into fresh culture medium.
  g. Next day, add 1 mg/mL G418 for stable cell line selection.
  h. Select cells with 1 mg/mL G418 for 2 weeks. During this process, replace with fresh selection medium containing 1 mg/mL G418 every 2–3 days.
  i. Select single colonies containing more than 50 cells to the 24-well plate to continuous expansion (Figures 2C and 2D, left panels).
  j. Collect (0.5–1)×10^6^ cells from each colony, extract cellular total RNAs and reverse-transcribed into cDNAs.
  k. Test for transgene expression using qRT–PCR to select positive colonies (Figures 2C and 2D, right panels).

**Table 1:** The sequences of shRNAs and primers

Oligonucleotide	Sequence (5′-3′)
Lv-NC_F	TGTTCTCCGAACGTGTCACGTTTCAAGAGAACGTGACACGTTCGGAGAACTTTTTTC
Lv-NC_R	TCGAGAAAAAAGTTCTCCGAACGTGTCACGTTCTCTTGAAACGTGACACGTTCGGAGAACA
Lv-324-1_F	TGCAGAGCTGGGATTACGTCTAAGATTTCAAGAGAATCTTAGACGTAATCCCAGCTCTGCTTTTTTC
Lv-324-1_R	TCGAGAAAAAAGCAGAGCTGGGATTACGTCTAAGATTCTCTTGAAATCTTAGACGTAATCCCAGCTCTGCA
Lv-324-2_F	TGAGAAATGCGCTGACAAATCTTAAATTCAAGAGATTTAAGATTTGTCAGCGCATTTCTCTTTTTTC
Lv-324-2_R	TCGAGAAAAAAGAGAAATGCGCTGACAAATCTTAAATCTCTTGAATTTAAGATTTGTCAGCGCATTTCTCA
p53 siRNA (si-p53)	Sense: 5′-GACUCCAGUGGUAAUCUACdtdt-3'; anti-sense: 5′-GUAGAUUACCACUGGAGUCdtdt-3′
LINC00324 Forward primer	CTACGGTTTCTGGTCAGCGT
LINC00324 Reverse primer	ACGACGGCAGCCATTACTTT
GAPDH Forward primer	ATCAATGGAAATCCCATCACCA
GAPDH Reverse primer	GACTCCACGACGTACTCAGCG

### Choice of primary antibodies

**Timing: ∼2 days**

The two primary antibodies must be raised in two different species and must bind with high specificity for the protein of interest. This section describes the procedure to identify the specificity of the SET and p53 antibodies considered to be used for PLA experiments.

3. Separate protein from U2OS and HepG2 cells extracts by SDS-PAGE and transfer onto the nitrocellulose membrane.
4. Block the membrane in 5% skim milk for 1 h at room temperature (22°C–26°C).
5. Remove the milk and incubate the membranes in antibody solutions of rabbit SET antibody (1:1000), mouse SET antibody (1:1000), or rabbit p53 antibody (1:1000) at 4°C for 12–18 h.
6. Wash the membrane in 1× TBST 3 times for 10 min each at room temperature (22°C–26°C).
7. Incubate the membrane with corresponding secondary antibodies for 1 h at room temperature (22°C–26°C).
8. Wash the membrane in 1× TBST 3 times for 10 min each at room temperature (22°C–26°C).
9. Develop the target band signals using an ECL solution and a ChemiDoc XRS detection system.

**Figure 3:**
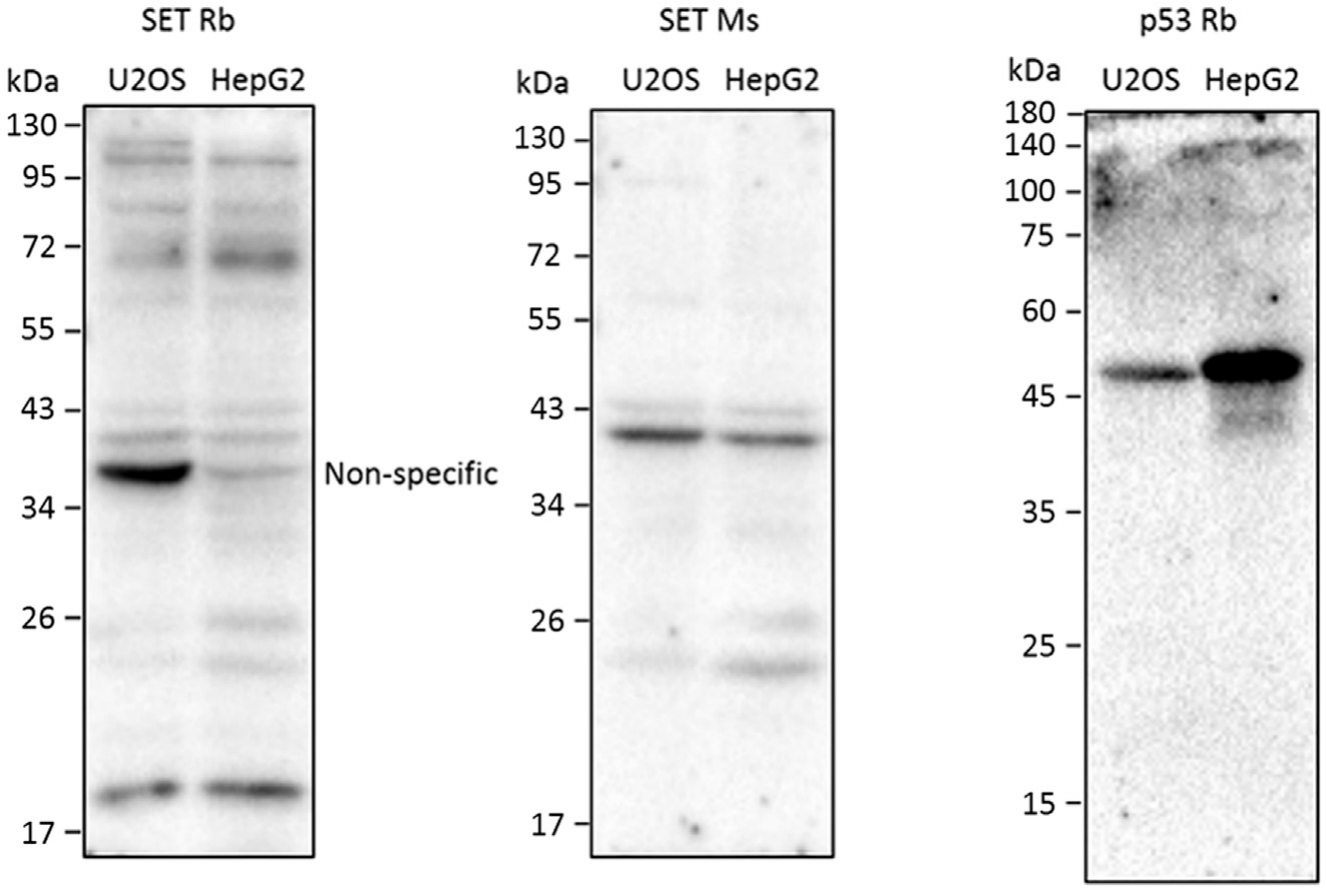
Identification of primary antibodies specificity Western blot analysis of SET rabbit antibody, SET mouse antibody and p53 rabbit antibody using cell extracts from U2OS and HepG2 cells. Rb, rabbit; Ms, mouse. These data are from the original Figure S5A in Zhang et al. (2023).^1^

**CRITICAL:** To assess p53-SET interaction, we tested the specificity of rabbit and mouse SET antibodies, and p53 antibodies. The mouse SET antibody and rabbit p53 antibody showed optimal specificity while rabbit SET antibody showed non-specific bands (Figure 3). We chose mouse SET antibody and rabbit p53 antibody for PLA experiment.

### Reagents preparation

**Timing: ∼30 min**

10. 1× PBS: Dilute 10× PBS in a 1:10 ratio in Milli-Q water.
11. 0.3% Triton X-100: Mix 1× PBS with 0.3% (vol/vol) Triton X-100.
12. 1× Wash buffer (Wash buffer A and B): Dissolve the content of 1 pouch of powdered buffer in Milli-Q water to a final volume of 1 L.
13. 0.01× Wash buffer B: Dilute 1× Wash buffer B in a 1:100 ratio in Milli-Q water.

***Note:*** Keep the solutions at room temperature (22°C–26°C) for short-term storage (one week or less). For long-term storage, store at 4°C.

## Key resources table

**Table:** 

REAGENT or RESOURCE	SOURCE	IDENTIFIER
**Antibodies**		

Rabbit anti-P53 (1:1,000 for WB, 1:100 for PLA)	Proteintech	Cat# 10442-1-AP; RRID: AB_2206609
Mouse anti-SET (1:1,000 for WB, 1:100 for PLA)	Santa Cruz	Cat# sc-133138; RRID: AB_2185628
Rabbit anti-SET (1:1,000 for WB)	Proteintech	Cat# 55201-1-AP; RRID: AB_10837360
Anti-mouse IgG, HRP (1:3,000)	GE Healthcare	Cat# NA931; RRID: AB_772210
Anti-rabbit IgG, HRP (1:5,000)	GE Healthcare	Cat# NA934; RRID: AB_772206

**Chemicals, peptides, and recombinant proteins**		

DAPI Fluoromount G	Yeasen	Cat# 36308ES11
G418	Gibco	Cat# 11811-031
Puromycin	Beyotime	Cat# ST551
Polybrene	GenePharma	N/A
Lipofectamine 2000	Invitrogen	Cat# 11668-019
Lipofectamine RNAiMAX	Invitrogen	Cat# 13778-150
Opti-MEM	Gibco	Cat# 11058-021
Duolink *in situ* wash buffers, fluorescence	Sigma-Aldrich	Cat# DUO82049
10× PBS	Solarbio	Cat# P1022
4% PFA	Solarbio	Cat# P1110
Triton X-100	Diamond	Cat# A110694-0500
DMEM	Corning	Cat# 10-013-CV
MEM	Gibco	Cat# 11090-081
FBS	VivaCell	Cat# C04001-500
Penicillin/Streptomycin	Gibco	Cat# 15140-122
NEAA	Gibco	Cat# 11140-050
GlutaMAX	Gibco	Cat# 35050-061
Sodium pyruvate	Gibco	Cat# 11360-070
Tris-base	BBI Solutions	Cat# A501492-0005
Tris-HCl	BBI Solutions	Cat# A610103-0250
Tween 20	Diamond	Cat# A100777-0500
NaCl	Sigma-Aldrich	Cat# S7653
Immobilon western HRP substrate	Millipore	Cat# WBKLS0500

**Critical commercial assays**		

TRIzol reagent	Invitrogen	Cat# 15596026
RevertAid First-Strand cDNA Synthesis kit	Thermo Fisher Scientific	Cat# K1622
SYBR Green master mix	Applied Biosystems	Cat# 4367659
Duolink *in situ* PLA probe anti-rabbit MINUS	Sigma-Aldrich	Cat# DUO92005
Duolink *in situ* PLA probe anti-mouse PLUS	Sigma-Aldrich	Cat# DUO92001
Duolink *in situ* detection reagents FarRed	Sigma-Aldrich	Cat# DUO92013

**Experimental models: Cell lines**		

HepG2 cells	Cell Bank of Chinese Academy of Sciences	SCSP-510
U2OS cells	Kept in laboratory	N/A
293T cells	Kept in laboratory	N/A

**Oligonucleotides**		

Sequences of shRNAs, see Table 1	Zhang et al.^1^	N/A
Sequences of siRNA, see Table 1	Zhang et al.^1^	N/A
Sequences of primers, see Table 1	Zhang et al.^1^	N/A

**Recombinant DNA**		

pcDNA3.1/myc-His-A (+) -LINC00324	Zhang et al.^1^	N/A
LV2 (U6/Puro)	GenePharma	N/A
pGag/Pol	GenePharma	N/A
pRev	GenePharma	N/A
pVSV-G	GenePharma	N/A

**Software and algorithms**		

Prism 8.0	GraphPad	https://www.graphpad.com
ImageJ	National Institutes of Health	https://imagej.nih.gov/ij/

**Other**		

Coverslips	WHB Scientific	Cat# WHB-24-CS
6-well plate	Costar	Cat# 3516
24-well plate	Costar	Cat# 3524
50 mL centrifuge tube	Corning	Cat# 430829
0.45 μm filter	Millipore	Cat# SLHV033RB
Nitrocellulose membrane	GE Healthcare	Cat# 10600002
Hydrophobic PAP pen	Gene Tech	Cat# GT1001
ChemiDoc imaging system	Bio-Rad	ChemiDoc XRS
Confocal laser scanning microscope	Leica Microsystems	TCS SP8

## Materials and equipment

**Table:** U2OS culture medium

Reagent	Final concentration	Amount
Dulbecco’s modified Eagle’s medium (DMEM)	N/A	445 mL
FBS	10% (v/v)	50 mL
Penicillin/Streptomycin	100 U/mL, 100 μg/mL	5 mL
**Total**	**N/A**	**500 mL**

**Table:** HepG2 culture medium

Reagent	Final concentration	Amount
Minimum Essential Medium (MEM)	N/A	430 mL
FBS	10% (v/v)	50 mL
Penicillin/Streptomycin	100 U/mL, 100 μg/mL	5 mL
GlutaMAX	2 mM	5 mL
sodium pyruvate	1 mM	5 mL
Nonessential amino acids (NEAA)	1%	5 mL
**Total**	**N/A**	**500 mL**

***Note:*** Store the culture medium at 4°C for 1 month. Bring the culture medium to room temperature (22°C–26°C) for 1 h or warm the medium at 37°C water bath for 30 min before use.

**Table:** 1× Wash buffer A: It can also be prepared in the lab as follows instead of which is provided as a powder from Sigma–Aldrich

Reagent	Final concentration	Amount
NaCl	0.15 M	8.8 g
Tris base	0.01 M	1.2 g
Tween 20	0.05%	0.5 mL
Milli-Q water	N/A	Up to 1 L
**Total**	**N/A**	**1 L**

Adjust the pH to 7.4. Filter the solution through a 0.22 μm filter. Store at 4°C for up to 1 year.

**Table:** 1× Wash buffer B: It can also be prepared in the lab as follows instead of which is provided as a powder from Sigma–Aldrich

Reagent	Final concentration	Amount
NaCl	0.1 M	5.84 g
Tris base	0.04 M	4.24 g
Tris-HCl	0.16 M	26 g
Milli-Q water	N/A	Up to 1 L
**Total**	**N/A**	**1 L**

Adjust the pH to 7.5. Filter the solution through a 0.22 μm filter. Store at 4°C for up to 1 year.

## Step-by-step method details

### Preparation of cells

**Timing: ∼72 h**

This section describes the procedure to prepare cells for proximity ligation assay (PLA).

1. Place the 14 mm round glass coverslips into a 24-well plate.

***Note:*** Coverslips should be clean and sterile.

2. Seed cells onto the coverslips in the 24-well plate.
3. Let cells stand for 15 min for adhesion and then incubate at 37°C in the CO_2_ incubator for 24 h.
4. For p53 knockdown, transfect 10 pmol siRNA for p53 into LINC00324 knockdown and overexpression U2OS cells using the Lipofectamine RNAiMAX transfection reagent according to manufacturer’s instructions (https://www.thermofisher.com/us/en/home/references/protocols/rnai-epigenetics-and-gene-regulation/rnai-protocol/lipofectamine-rnaimax.html). Add 10 pmol si-p53 to 50 μL Opti-MEM medium and 1.5 μL Lipofectamine RNAiMAX reagent to 50 μL Opti-MEM medium. Combine both solutions and incubate for 10–20 min at room temperature (22°C–26°C). Add the complexes to each well containing cells. Mix gently by rocking the plate back and forth. Incubate the cells at 37°C in a CO_2_ incubator. After 48 h growing, subject the cells to fixation.

***Note:*** The optimal cell confluence is 50%–70% at the time of fixation.

### Cell pretreatment

**Timing: ∼2.5 h**

This section describes the steps for cell fixation, permeabilization and blocking.

5. Fixation.
  a. Remove medium, wash cells with 1× PBS for 5 min at room temperature (22°C–26°C).
  b. Fix cells with 4% PFA for 20 min at room temperature (22°C–26°C).

**Pause point:** Fixed cells can be stored in 1× PBS at 4°C for several days. The plates should be prevented from drying out.

***Note:*** The fixation time may vary according to the cell type. Methanol is an alternative fixing reagent. The best choice of fixation may vary depending on your sample type, antibodies, and the protein target you want to study.

6. Permeabilization.
  a. Wash cells with 1× PBS 3 times for 5 min each at room temperature (22°C–26°C).
  b. Permeabilize with 1× PBS with 0.3% Triton X-100 for 20 min at room temperature (22°C–26°C).
7. Blocking.
  a. Wash cells with 1× PBS 3 times for 5 min each at room temperature (22°C–26°C).
  b. Put the coverslips on slides. Draw a hydrophobic circle along the coverslips’ edges with a hydrophobic PAP pen to delimit the reaction area.
  c. Add 60 μL of Duolink Blocking Solution to each sample.
  d. Incubate the slides in a humidity chamber for 1 h at 37°C.

**Pause point:** Blocking can be run for 12–18 h at 4°C.

**CRITICAL:** Be sure to cover the entire sample with all solutions. Do not allow the cells to dry before adding the antibody.

### Primary antibodies incubation

**Timing: ∼18 h**

This section describes the steps for primary antibodies incubation.

8. Dilute primary antibodies anti-p53 (rabbit) and anti-SET (mouse) in a 1:100 ratio using Duolink Antibody Diluent.

***Note:*** Dilute your primary antibody to a suitable concentration in your custom antibody diluent. If using two primary antibodies, dilute the two antibodies in the same diluent.

9. Remove the blocking solution from the coverslips and add 60 μL of the primary antibodies solution to each sample.

***Note:*** Aspirate off the blocking solution as much as possible to avoid dilution of the primary antibodies.

10. Incubate the slides in a humidity chamber for 12–18 h at 4°C.

**CRITICAL:** Do not allow the cells to dry. Make sufficient solutions for all samples.

The following PLA steps are referred to the Duolink PLA Fluorescence Protocol from Sigma-Aldrich with minor modifications.

### Duolink PLA probe incubation

**Timing: ∼1.5 h**

This section describes the steps for probe hybridization.

11. Dilute the two 5× Duolink PLA probe and mix Anti-Mouse PLUS and Anti-Rabbit MINUS PLA probes at 1:5 dilution in the Duolink Antibody Diluent.
12. Remove the primary antibody solution and wash the slides with 50 mL 1× Wash Buffer A 2 times for 5 min each on a shaker at room temperature (22°C–26°C).

***Note:*** Bring the wash buffer to room temperature (22°C–26°C) before use.

13. Remove the excess wash buffer and add 60 μL of the PLA probe mixture solution to each sample.
14. Incubate the slides in a pre-heated humidity chamber for 1 h at 37°C.

***Note:*** Do not allow the cells to dry. Make sufficient solution for all samples.

### Ligation

**Timing: 1 h**

This section describes the steps for oligonucleotide ligation.

15. Remove the PLA probe solution, wash the slides with 50 mL 1× Wash Buffer A 2 times for 5 min each at room temperature (22°C–26°C).
16. Dilute the 5× Duolink Ligation buffer in a 1:5 ratio in Milli-Q water.

***Note:*** The buffer contains DTT that may precipitate during freezing. Gently pipette to dissolve.

17. Add ligase to the 1× Ligation buffer from step 16 in a 1:40 ratio and mix.

***Note:*** Ligase should stay on ice. Add the enzyme to the reaction mix immediately before use.

18. Remove the excess wash buffer and add 60 μL of the Ligase-Ligation solution to each sample.
19. Incubate the slides in a humidity chamber for 30 min at 37°C.

### Rolling circle amplification (RCA)

**Timing: ∼2 h**

This section describes the steps for rolling circle amplification.

20. Remove the Ligase-Ligation solution, wash the slides with 50 mL 1× Wash Buffer A 2 times for 5 min each at room temperature (22°C–26°C).
21. Dilute the 5× Amplification Far Red buffer in a 1:5 ratio in Milli-Q water.

***Note:*** Amplification buffer is light-sensitive. Make sure to protect from light from this step.

22. Add Polymerase to the 1× Amplification buffer from step 21 in a 1:80 ratio and mix.

***Note:*** Polymerase should stay on ice. Add the enzyme to the reaction mix immediately before use.

23. Remove the excess wash buffer and add 60 μL of the Polymerase-Amplification solution to each sample.
24. Incubate the slides in a humidity chamber for 100 min at 37°C.

### Final washes

**Timing: ∼25 min**

This section describes the steps for washing after amplification.

25. Remove the Polymerase-Amplification solution, wash the slides with 50 mL 1× Wash Buffer B 2 times for 10 min each at room temperature (22°C–26°C).
26. Wash the slides with 50 mL 0.01× Wash Buffer B for 1 min at room temperature (22°C–26°C).

***Note:*** Make sure the slides are protected from light during the procedure.

### Mounting and imaging

**Timing: ∼30 min to 3 h**

This section describes the steps for coverslips mounting and image acquisition.

27. Remove the excess wash buffer, air dry in the dark.

***Note:*** a paper towel can be used to carefully absorb the excess buffer.

28. Drop 5 μL DAPI Fluoromount G on a microscope glass slide. Put coverslips upside down on the slide carefully to make sure the mounting solution is spread all over.

***Note:*** Avoid air bubbles during the mounting process. You can also use the Duolink In Situ Mounting Medium with DAPI from Sigma-Aldrich as an alternative.

29. Place the slides in the dark for 15 min at room temperature (22°C–26°C) to dry before viewing using a confocal microscope (LEICA TCS SP8).

***Note:*** Use clear nail polish or neutral balsam to seal all sides of coverslips for long-time storage.

30. Acquire images using at least a 20× objective.

**Pause point:** The slides can be stored at 4°C for 3–4 days or at –20°C for longer time before imaging.

**CRITICAL:** Make sure the images in the experimental and control groups acquired in the same parameters for proper comparison.

## Expected outcomes

**Figure 4:**
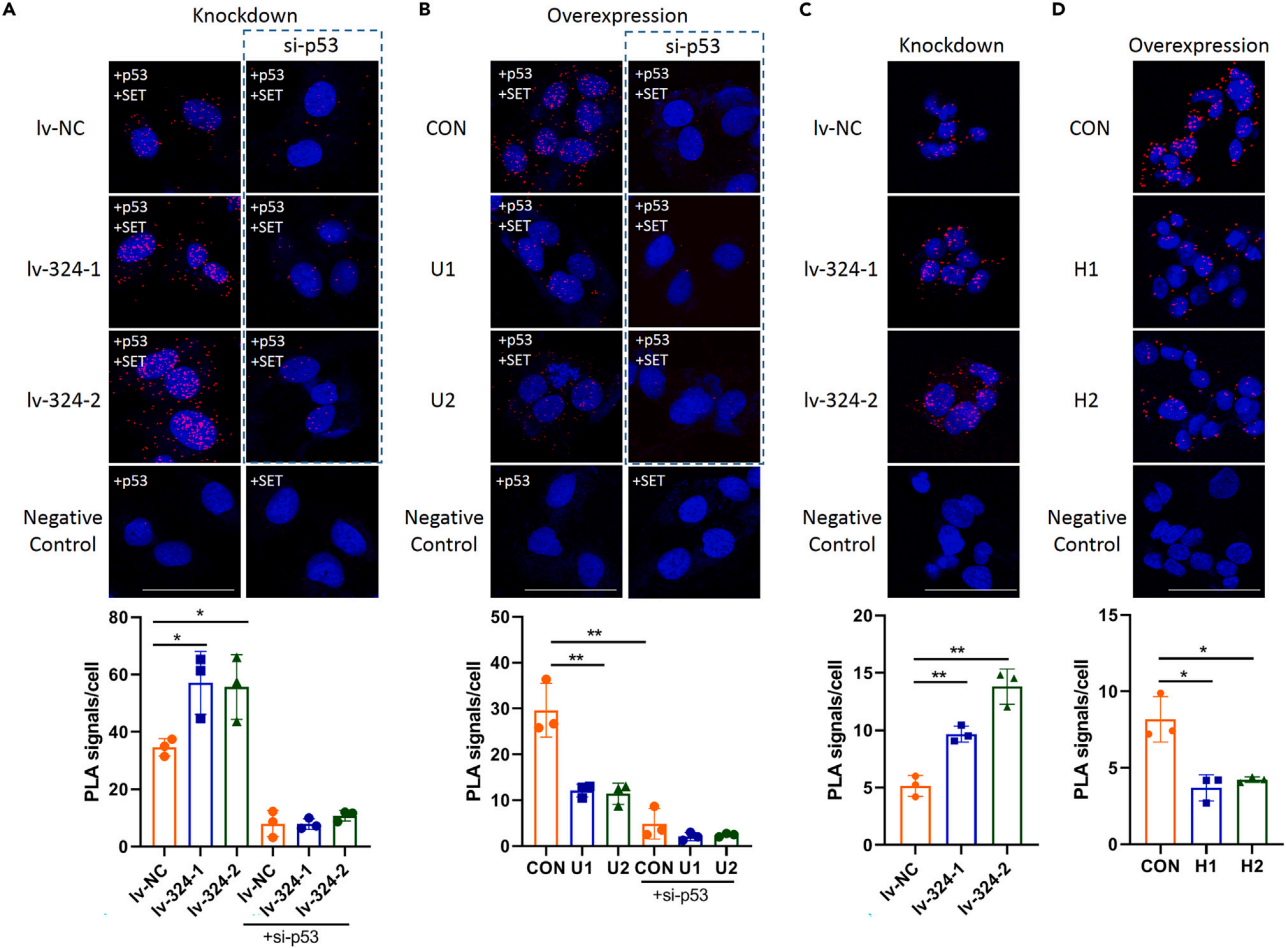
Confocal images of *in situ* PLA show that LINC00324 disrupts the p53-SET interaction The data of Figure 4C and 4D are from the original Figures S5B and S5C in Zhang et al. (2023).^1^ (A and B) Duolink PLA detected the interaction between p53 and SET in U2OS cells stably expressing the shRNAs lv-NC, lv-324-1, and lv-324-2 (A) and in U2OS control cells transfected with empty vector (CON) and U2OS cells stably expressing ectopic LINC00324 (U1 and U2) (B). siRNA against p53 and single antibody p53 or SET were performed as negative controls. (C and D) Duolink PLA detected the interaction between p53 and SET in HepG2 cells stably expressing the shRNAs lv-NC, lv-324-1, and lv-324-2 (C) and in HepG2 control cells transfected with empty vector (CON) and HepG2 cells stably expressing ectopic LINC00324 (H1 and H2) (D). The average number of PLA dots from three random fields per coverslip was quantitated and presented in the bar diagram. Scale bar, 50 μm ∗p < 0.05, ∗∗p < 0.01, which were calculated using two-tailed Student’s t test. The data are presented as mean ± SD.

The PLA signal recognized as fluorescent spots should be produced by successful PLA if the two proteins are closer than 40 nm.^7^^,^^8^ In this protocol we performed PLA in U2OS and HepG2 cells to determine the effects of LINC00324 on p53-SET interaction by comparing fluorescent spots between the controls and LINC00324 knockdown or overexpression cells (Figures 4A–4D). PLA signals should not be detected in the sample in which the single antibody was added to (Negative control). The results demonstrated that the PLA spots were increased in LINC00324 knockdown U2OS and HepG2 cells (Figures 4A and 4C) but were suppressed in LINC00324 overexpression cells (Figures 4B and 4D). These results indicate that LINC00324 interrupts the p53-SET interaction.

## Quantification and statistical analysis

Open the images using Image J software. Count the number of PLA spots and nuclei manually using “Multi-points” tool. The average PLA spots of each cell were calculated by dividing the number of PLA spots of all cells by the number of nuclei in the visual field. Take the average of the three random fields. Perform student’s t-test analysis using GraphPad Prism 8.0 (Figures 4A–4D).

## Limitations

First, there exist off-target effects for the shRNA-mediated knockdown. One should choose at least two shRNA sequences targeting the different locations of an lncRNA. Second, it is difficult to get effective shRNAs to knock down a target lncRNA in some conditions. More shRNAs sequences should be designed in order to select effective fragments. Third, the long-term antibiotic selection may affect cell growth or even lead to cell differentiation resulting from shRNA-mediated knockdown or other indirect effects. One should be careful that the observed effects of an lncRNA on PPIs were caused from an indirect effect. Similar conditions may appear for the overexpression stable cells. Transient siRNA-mediated knockdown or overexpression experiments can be used to confirm the results, albeit at a lower expression efficiency compared with stable cells. Finally, an unspecific signal may appear if the primary antibody recognizes non-specific proteins. Try several antibodies from different source to determine which one is fit for the experiment. If the specificity of signals was suspicious, one may perform siRNA-mediated target protein knockdown to confirm the specificity.

## Troubleshooting

### Problem 1

The density of cells is too low or too high to produce high-quality images (Related to preparation of cells: step 2–3).

### Potential solution

Proper cell seeding density should be determined according to different cell growth rate of various cells. Cells are seeded onto coverslips and the plates may stay in the clean bench for 30 min at room temperature (22°C–26°C) before moved to the incubator to avoid cells clumping.

### Problem 2

Cells are not adherent to the coverslips (Related to preparation of cells: step 1).

### Potential solution

Coverslips need to be coated with collagen or Poly-lysine.

### Problem 3

Images have high background signals (Related to step-by-step method details: step 7–10).

### Potential solution

This may be related to unspecific binding of primary antibodies, insufficient blocking, insufficient washing, and drying of samples. You can decrease antibody concentration or use other primary antibodies. Prolong the blocking time. Increase washing time and/or add more washing steps. Add adequate liquid to prevent from drying out (recommended reaction volume: 40 μL per 1 cm^2^ coverslips).

Dust, salt or precipitates in buffers may cause high fluorescent particles. Sterile filter all solutions. Use new solutions and washing jars. Wash cells at least twice to completely remove the culture medium before fixation.

### Problem 4

There are no or low PLA signals even though other methods have shown the target proteins are interacting. (Related to step-by-step method details: step 8–30).

### Potential solution

Optimize antibody concentration, reaction temperature and time by performing IF or IHC experiment. Make sure to completely remove the wash buffer before adding antibodies, the ligation and amplification solutions. Reduce the wash time. Ensure all the solutions are not expired.

Image acquirement may not be proper. Use appropriate settings during imaging.

### Problem 5

There is no PLA signal in the nucleus, but exist in the rest of the cell, even though the proteins of interest are in the nucleus. (Related to step-by-step method details: step 6)

### Potential solution

This may be related to insufficient permeabilization. You can increase the Triton X-100 concentration and the permeabilization time.

Nuclear protein interactions can be masked by nuclear crowding effects. A pre-extraction step like the employment of Cytoskeletal (CSK) buffer can be used.

## Resource availability

### Lead contact

Further information and requests for resources and reagents should be directed to and will be fulfilled by the lead contact, Dawei Li (daweili@suda.edu.cn).

### Materials availability

This research did not produce any new unique reagents.

### Data and code availability

This study did not produce any unique dataset or code.
